# Functional Characterization Improves Associations between Rare Non-Synonymous Variants in *CHRNB4* and Smoking Behavior

**DOI:** 10.1371/journal.pone.0096753

**Published:** 2014-05-07

**Authors:** Gabe Haller, Ping Li, Caroline Esch, Simon Hsu, Alison M. Goate, Joe Henry Steinbach

**Affiliations:** 1 Departments of Psychiatry and Genetics, Washington University, St. Louis, Missouri, United States of America; 2 Department of Anesthesiology, Washington University, St. Louis, Missouri, United States of America; 3 Department of Psychiatry, Washington University, St. Louis, Missouri, United States of America; 4 Department of Anesthesiology and the Taylor Family Institute for Innovative Psychiatric Research, Washington University, St. Louis, Missouri, United States of America; University of Michigan, United States of America

## Abstract

Smoking is the leading cause of preventable death worldwide. Accordingly, effort has been devoted to determining the genetic variants that contribute to smoking risk. Genome-wide association studies have identified several variants in nicotinic acetylcholine receptor genes that contribute to nicotine dependence risk. We previously undertook pooled sequencing of the coding regions and flanking sequence of the *CHRNA5, CHRNA3, CHRNB4, CHRNA6* and *CHRNB3* genes and found that rare missense variants at conserved residues in *CHRNB4* are associated with reduced risk of nicotine dependence among African Americans. We identified 10 low frequency (<5%) non-synonymous variants in *CHRNB4* and investigated functional effects by co-expression with normal α3 or α4 subunits in human embryonic kidney cells. Voltage-clamp was used to obtain acetylcholine and nicotine concentration–response curves and qRT-PCR, western blots and cell-surface ELISAs were performed to assess expression levels. These results were used to functionally weight genetic variants in a gene-based association test. We find that there is a highly significant correlation between carrier status weighted by either acetylcholine EC_50_ (β = −0.67, r^2^ = 0.017, P = 2×10^−4^) or by response to low nicotine (β = −0.29, r^2^ = 0.02, P = 6×10^−5^) when variants are expressed with the α3 subunit. In contrast, there is no significant association when carrier status is unweighted (β = −0.04, r^2^ = 0.0009, P = 0.54). These results highlight the value of functional analysis of variants and the advantages to integrating such data into genetic studies. They also suggest that an increased sensitivity to low concentrations of nicotine is protective from the risk of developing nicotine dependence.

## Introduction

Nicotinic acetylcholine receptors (nAChRs) are pentameric ligand-gated ion channels formed from numerous combinations of receptor subunits, each encoded by a separate gene. Neuronal nAChR α subunits are encoded by eight genes in mammals (*CHRNA2*-*A7*, *CHRNA9-10*) and the β subunits by three genes (*CHRNB2*-*B4*). The α7, α9 and α10 subunits can form functional receptors without incorporating a β subunit, while a minimum of two α and two β subunits (plus one additional subunit) are required to form functional heteromeric receptors. Certain combinations of receptor subunits are more common in the central nervous system (CNS), and there is regional specificity with regard to subtype expression in the mammalian CNS [1]. The expression of the α3β4* subtype (the asterisk denotes any one of multiple accessory subunits), for instance, is limited for the most part to autonomic and sensory ganglia, medial habenula and the interpeduncular nucleus (IPN), while α4β2* receptors can be found almost ubiquitously throughout the brain [2], [3], [4].

Variants in or near nicotinic acetylcholine receptor genes have been found to be associated with nicotine related behavior in humans. A non-synonymous change (rs16969968; α5 D398N) in *CHRNA5* is the most strongly associated single nucleotide polymorphism (SNP) in several genome-wide association studies (GWAS) of smoking quantity [5], [6], with the N398 variant associated with increased risk. Additionally, variants near *CHRNA5,* that alter *CHRNA5* mRNA expression *in vivo*, alter risk for both nicotine and alcohol dependence [7], [8]. A group of common SNPs near the *CHRNB3-CHRNA6* gene cluster also are associated with cigarette consumption in a recent GWAS [5]. Sequencing of the neuronal nicotinic receptor genes in cohorts of nicotine dependent cases and controls has also found associations between variants in three nicotinic receptor genes, *CHRNA3, CHRNA4* and *CHRNB4,* with the risk for nicotine dependence [9], [10], [11]. These findings indicate that the properties of nicotinic receptor subunits are strongly associated with the risk of developing nicotine dependence, but do not provide insights into the possible mechanisms for the association.

We have provided evidence that rare variants in *CHRNB4* identified in a deep resequencing study of a cohort of nicotine-dependent and control subjects were associated with reduced risk of developing nicotine dependence [11]. However, this association was based simply on the presence or absence of selected variants, without considering the possible functional effects a variant might have. In the present study we determine whether information from *in vitro* tests of functional consequences of non-synonymous coding variants can significantly improve the association between genotype and phenotype. We report the functional impact of rare variants in *CHRNB4*, and results that demonstrate that incorporating information on the functional consequences can improve the association between genotype and the complex behavioral phenotype of nicotine dependence. Furthermore, the results suggest that an increased response to low concentrations of nicotine may reduce the risk of developing nicotine dependence.

## Materials and Methods

### Ethics Statement

De-identified data from the Collaborative Genetic Study of Nicotine Dependence (COGEND) were used. All participants in COGEND provided written informed consent for genetic studies and agreed to share their DNA and phenotypic information for research purposes. The Washington University Human Research Protection Office granted approval for data to be used for this study.

### Generation and Expression of Constructs

Full length coding sequences for the human nicotinic α3 (NP_000734.2) and β4 (NP_000741.1) subunits were kindly provided by Dr. J. Lindstrom (University of Pennsylvania, Philadelphia, PA). Subunits were sub-cloned into the pcDNA3 vector (Life Technologies, Grand Island NY). The FLAG epitope [DYKDDDDK] was introduced into α4 between the 6 and 7 positions of the mature polypeptide using QuikChange (Stratagene, San Diego, CA). Mutations that produced the variants were also introduced using the QuikChange kit. For each construct the entire subunit coding region was sequenced to verify that only the desired mutation had been introduced.

### Cell Culture and Transfection

HEK 293 cells (American Tissue Culture Collection, Gaithersburg, MD) were maintained in a mixture of Dulbecco’s modified Eagle’s medium (DMEM) and Ham’s F12 (1∶1, also containing 2 mM L-glutamine and 15 mM HEPES), with 10% fetal bovine serum (Hyclone, Logan, UT), penicillin (100 units/ml) and streptomycin (100 ug/ml) in a humidified atmosphere containing 5% CO_2_ at 37°C. Cells were re-plated in the same medium the day before transfection.

For physiological and cell surface ELISA studies, subunits were transfected at a 1∶1 mass ratio using Effectene (Qiagen, Valencia, CA) according to the manufacturer’s instructions. Briefly, 3 µg of cDNA per well of a 24-well dish was mixed with the Enhancer and the Effectene Transfection Reagent. The cells were incubated with the mix for 6 to 18 h. Electrophysiological experiments were performed on either the second or third day after transfection, while ELISAs were performed on the third day after transfection.

For mRNA expression and protein expression experiments, cells were seeded into 6-well polylysine-coated plates and cultured in DMEM supplemented with 10% fetal bovine serum (FBS), 2 mM L-glutamine, and penicillin/streptomycin. Cells were transfected using Lipofectamine 2000 (Life Technologies, Carlsbad CA) according to the manufacturer’s protocol incubated in a humidified atmosphere containing 5% CO_2_ at 37°C.

### mRNA Expression

To measure *CHRNB4* mRNA production HEK 293 cells were transiently transfected with a negative control (empty pcDNA3), or wild-type α3 plus wild-type or variant β4 constructs. After two days of growth, RNA was extracted from cell lysates with an RNeasy kit (Qiagen). Extracted RNA (10 ug) was then converted to cDNA using the High Capacity cDNA Reverse Transcriptase kit (Life Technologies). *CHRNB4* mRNA expression was then measured using SYBRgreen (Life Technologies, Carlsbad, CA) using one primer spanning the boundary between exons 3 and 4 and another primer spanning the boundary between exons 4 and 5 to ensure only cDNA was amplified.

### Western Blots

To measure β4 protein levels in transiently transfected HEK293 cells, we performed western blots on cell lysates using a poly-clonal β4 antibody generously provided by Dr. Cecilia Gotti (CNR Institute of Neuroscience, Pisa, Italy). After two days of growth, each well of the 6-well plate was used to create a cell lysate. Cells were lysed with 150 µl of lysis buffer (50 mM Tris (pH 6.8), 150 mM NaCl, 2 mM EDTA, 0.25% NP40, 1% TritonX). Total protein concentration was then measured by the bicinchoninic acid (BCA) method (Thermo Fisher Scientific, Waltham MA) for each cell lysate. 20 µg of protein from each lysate was incubated at 95°C for 5 min in 1× Laemmli buffer (0.25 M Tris (pH 6.8), 8% SDS, 40% glycerol, 0.01% bromophenol blue dye and 20% β-mercaptoethanol). Denatured samples were loaded onto a 4–20% Criterion (Bio-Rad, Hercules, CA) gel in TG-SDS buffer (0.01% SDS, 25 mM Glycine, 2.5 mM Tris (pH 6.8)) and run at 125 V for 90 min. Protein in the gel was transferred to a nitrocellulose membrane in TG-SDS buffer containing 20% methanol overnight at 4°C. Blots were incubated in TG-SDS containing 4% powdered milk for 25 min at room temperature, then incubated at room temperature with a primary β4 polyclonal antibody for 90 min. The blots were then rinsed 3× with phosphate-buffered saline (PBS; 137 mM NaCl, 2.7 mM KCl, 4.3 mM Na_2_HPO_4_, and 1.4 mM KH_2_PO_4_, pH 7.3) containing 1% Triton-X for 5 min, incubated with a horseradish peroxidase conjugated secondary antibody (Thermo Fisher Scientific), washed 3× with PBS containing 1% Triton-X for 5 min and finally incubated with the horseradish peroxidase substrate 3, 3, 5, 5, 0-tetramethylbenzidine. Images were taken allowing for 5 min of exposure using a Syngene western blot imager (Syngene, Frederick, MD).

For digestion with PNGase, 1 µl of 10× glycoprotein denaturing buffer was added to 20 µg of protein from each lysate and incubated at 100°C for 10 min. Subsequently, 2 µl 10× G7 Reaction Buffer (NEB, Ipswich, MA), 2 µl 10% NP40 (NEB), H_2_O and 2 µl PNGaseF (NEB) were then added to reach a total reaction volume of 20 µl. This reaction was then incubated at 37°C for 1 h. Upon completion of the PNGase reaction, the resultant solution was run on western blots as described above.

### Cell-Surface ELISA

Surface ELISA assays were performed as described previously [12]. Briefly, cells were plated in 24 well tissue culture plates at about 100,000 cells/well. The next day cells were transfected as described. In each experiment, a negative control (empty pcDNA3) and a positive control (wild-type subunits) were performed. Five wells were transfected with each subunit combination in each experiment; 3 were used for ELISA and 2 for a protein assay. For the ELISA assay, cells were rinsed in PBS then blocked for 30 min at room temperature with 4% powdered milk in PBS (milk-PBS). To detect α3 mAb 35 was used as primary antibody (Sigma-Aldrich, St. Louis MO; diluted 1∶400 in milk-PBS) for 1 h at room temperature. Cells were then washed twice with milk-PBS and incubated with anti-rat IgG peroxidase-conjugated goat antibodies (Sigma-Aldrich, 1∶100 dilution in milk/PBS) for 1 h. To detect FLAG-tagged α4, cells were incubated with M2 antibody (Sigma-Aldrich, 2 µg/ml in milk-PBS) as described and then incubated with sheep anti-mouse IgG peroxidase-conjugated antibodies (Sigma-Aldrich; 1∶100 dilution in milk/PBS) for 1 h. Cells were washed with MPBS twice and PBS twice, then assayed using the 1-Step Ultra TMB-ELISA kit (Thermo Scientific). Absorbance was read at 405 nm using a microplate reader (iMark, Bio-Rad, Hercules, CA). Total cell protein was assayed from wells that had been maintained in milk-PBS, then washed twice with PBS before assay by a BCA method. For each experiment, the ELISA signal was obtained from triplicate wells and the cell protein from duplicate wells.

The surface ELISA data were analyzed as follows. The machine background was subtracted from each OD reading, then the OD readings were divided by the protein for that subunit combination. The normalized value for the negative control (pcDNA3) for that experiment was then subtracted from all values. Finally, to control for variation in expression between experiments, the subtracted expression levels were normalized to the positive control (wild-type) value for that experiment. The final value gives an estimate of the relative expression where a value of 1 indicates identical expression to wild-type subunits.

### Whole-cell Patch Clamp

Cells were plated in 35 mm tissue culture dishes, and maintained and transfected as described. For recordings, cells were rinsed with recording bath solution (140 mM NaCl, 5 mM KCl, 1 mM MgCl_2_, 2 mM CaCl_2_, 10 mM glucose, and 10 mM HEPES, pH 7.4) and cells expressing high levels of surface receptors were identified by a bead-binding technique [13]. We used mAb35 (Sigma-Aldrich) to identify α3 and mAb299 for α4 (Sigma-Aldrich). Antibody was adsorbed to immunobeads with a covalently attached secondary antibody (Life Technologies). The cells were incubated with a suspension of beads for 5 to 10 min with gentle shaking, and cells expressing surface receptors were identified from the presence of beads bound to the cell. This greatly enhanced our ability to identify cells expressing measurable numbers of receptors. The FLAG-tagged α4 subunit was not used in physiological experiments.

Macroscopic currents were recorded using whole-cell voltage clamp as described [13]. The pipette (intracellular) solution contained 140 mM CsCl, 4 mM NaCl, 4 mM MgCl_2_, 0.5 mM CaCl_2_, 5 mM EGTA, and 10 mM HEPES, pH 7.4. The drugs were applied through the bath using an SF-77B fast perfusion stepper system (Warner Instruments, Hamden, CT). Currents were recorded using an Axopatch 200 amplifier (Molecular Devices, Union City, CA). The cells were clamped at −60 mV and all experiments were carried out at room temperature (20–23°C). The current traces were low-pass filtered at 2 kHz and digitized at 10 kHz. The analysis of whole-cell currents was carried out using the pClamp 9.0 software package (Molecular Devices).

The basic parameter measured was the peak response to a given concentration of acetylcholine (ACh) or nicotine. Four sec pulses of agonist were applied at intervals of 30 sec with continuous application of bath solution between pulses to allow recovery from desensitization. Concentration-response relationships were obtained by applying pulses of different agonist concentration. The data from a cell were analyzed by fitting with the Hill equation: Y(X) = a(1/(1+(X/EC_50_)^n^)) where Y(X) is the response to a concentration X, and the parameters a (maximal response), EC_50_ (concentration giving half-maximal response) and n (Hill coefficient) were determined by non-linear regression using SigmaPlot (Systat Software, Chicago, IL). To combine data, the relationship for each cell was scaled by the fit maximum. In some cases, it was clear that high concentrations of agonist produced a reduced response, likely due to open-channel block by the agonist [14], [15], [16]. Accordingly, responses to high concentrations that produced a lower response than responses to lower concentrations were not included in the fit.

Concentration-response data for ACh and nicotine were obtained on different cells, to minimize the duration of whole-cell recording. Previous work has demonstrated that there can be changes in the peak response and/or the desensitization properties of neuronal nicotinic receptors over the time of whole-cell recording [15], [17]. Accordingly, each cell was tested with a high concentration of ACh (usually 1 mM), and this value was used to normalize the nicotine concentration-response relationship to the overall averaged ACh concentration-response relationship for that particular subunit combination. Similarly, some cells were tested with single applications of a high concentration of ACh and a high concentration of nicotine to obtain estimates for relative maximal responses. In this case, the relative maximal response to nicotine was normalized to both the mean concentration-response data for nicotine and for ACh for that subunit combination.

Desensitization was estimated from the ratio of the current at the end of the 4 sec pulse of agonist to the peak current during the application. Studies of desensitization can be complicated by the presence of additional receptor processes (such as channel block). Accordingly, the measurements were performed using the concentrations closest to the half-maximal concentration of agonist to avoid channel block. Neuronal nicotinic receptors show evidence for multiple, kinetically distinct, forms of desensitization [15], [17] that may exhibit both agonist and concentration dependence in prevalence. Accordingly, our measurement provides a rough estimate of the rate of accumulation of desensitized receptors. We did not examine recovery from desensitization. To avoid over definition of the phenomenon, we will call this measurement “sag” in the text.

The cells used for physiological studies all were selected on the basis that they expressed receptors on the cell surface. Accordingly the average maximal response to acetylcholine will not be representative of the total cell population. Therefore, we adopted the relative cell surface ELISA signal as the assay for numbers of surface receptors for the different receptors expressed.

### Drugs, Data Presentation and Statistics

Unless otherwise noted all chemicals used were obtained from Sigma-Aldrich.

Data are presented as mean ± SE (number of observations). For ELISA studies results from each experiment constituted one observation; data were obtained in triplicate in each experiment. For physiological results each cell constituted an observation (i.e. EC_50_ or relative maximal response).

Parameters from fitting the Hill equation were excluded from analysis if the standard error of any fit parameter for that cell (as estimated by the fitting program) was 60% or more of the best fitting value. Data from ELISA experiments were excluded if the expression normalized to protein for the positive control (wild-type subunits) did not differ from that for the negative control (pcDNA3) with a probability of less than 0.05 (two-tailed t-test), indicating a failed transfection.

Basic statistical computations were made with Excel (Microsoft, Redmond WA). ANOVA was performed using Stata (StataCorp, College Station TX). Figures were prepared with SigmaPlot (Systat Software, San Jose CA).

The homology model was made by threading the α3 and β4 subunits onto the C and D subunits respectively of the *C. elegans* glutamate-activated Cl^-^ channel X-ray crystal structure ([18]; PDB 3RHW) using the SWISS-MODEL web tool (http://swissmodel.expasy.org/). Structures were visualized and displays generated using Chimera 1.6.2 (http://www.cgl.ucsf.edu/chimera).

### Samples and Phenotype

DNA samples were collected as part of the Collaborative Genetic Study of Nicotine Dependence (COGEND). All individuals were current or former smokers who had smoked at least 100 cigarettes in their lifetime and underwent a semi-structured interview, which assessed smoking behavior, other substance use and comorbid psychiatric conditions. The COGEND sample includes 710 African Americans (461 nicotine dependent (ND) cases and 249 smokers with no symptoms of dependence (controls). Nicotine dependent cases have a Fagerström Test of Nicotine Dependence (FTND) [19] score ≥4 while controls have an FTND ≤1. In all cases lifetime maximum FTND score was used. One of the questions within the FTND asks “How many cigarettes did you smoke per day when you were smoking the most?” This value, cigarettes per day (CPD), was used in our analyses assessing whether functional characterization of alleles could improve observed associations. A total of 352 African Americans were sequenced in the original study. Follow up genotyping of SNPs identified and validated in the sequenced individuals was done in the remaining portion of the COGEND African American sample (710 individuals total).

We focused our attention on the African-American population for the following reasons. In our initial report [11] in which we analyzed the association between carrier status for variants at four conserved sites (T91I, G296S, T375I and M456V), there was a significant association of carrier status with the control group for the AA but not EA population. Further, the AA population harbored 10 rare variants, while the EA population only had 4. This suggested that the AA population was more suitable for the analysis.

The Collaborative Genetic Study of Nicotine Dependence is a collaborative research group and part of the National Institute on Drug Abuse (NIDA) Genetics Consortium (http://www.ncbi.nlm.nih.gov/projects/gap/cgi-bin/study.cgi?study_id=phs000092.v1.p1). Subject collection was supported by NIH grant P01 CA89392 (PI - L Bierut) from the National Cancer Institute. Phenotypic and genotypic data are stored in the NIDA Center for Genetic Studies (NCGS) at http://zork.wustl.edu/under NIDA Contract HHSN271200477451C (PIs J Tischfield and J Rice).

### Ethics Statement

De-identified data from the Collaborative Genetic Study of Nicotine Dependence (COGEND) were used. All participants in COGEND provided written informed consent for genetic studies and agreed to share their DNA and phenotypic information for research purposes.

### Association Analysis

Association analyses were performed in R (www.r-project.org) using linear regression incorporating age and sex as covariates. In order to ensure that associations were not affected by population stratification, we calculated principal components (PCs) with ∼3700 SNPs spread out across the genome [20] using EIGENSTRAT [21] and included the first two PCs as covariates in all analyses. Carrier status was tested in a linear regression against logCPD with age, sex, PC1 and PC2 as covariates. CPD values were log transformed to approximate a normal distribution. To analyze carrier status, individuals were coded as either 0 or 1 depending on whether they carried 0 or ≥1 missense variants at any position in *CHRNB4* [22]. To test the effect of variants occurring at “conserved” residues (vertebrate phyloP score >2; [23]), only individuals with such variants were coded as 1. To analyze the function-weighted carrier status, individuals were coded with the normalized parameter value for the tested parameter (e.g. (ACh EC_50_, nicotine EC_50_, nicotine efficacy, response to low nicotine and cell-surface expression) for the particular variant the individual carried. These values were then used as the predictor in a linear regression using age, sex, and the first two principal components (PC1 and PC2) as covariates. As described in the Results, data for variants occurring in only one individual were included by weighting them by the parameter value of the non-singleton variant or wild-type with the closest parameter estimate.

Permutations for each of the significant parameters were performed in R by randomly assigning one of the measured parameter values to each of the variants without replacement. To approximate the experimentally observed probability that the association is significant we performed either 10,000 or 20,000 permutations. With these numbers of permutations a single more significant result in the permutations would correspond to a frequency of 0.0001 or 0.00005 that the experimental observation would arise by chance.

## Results

### Location of the Variants in a Homology Model of the Receptor

**Figure 1:**
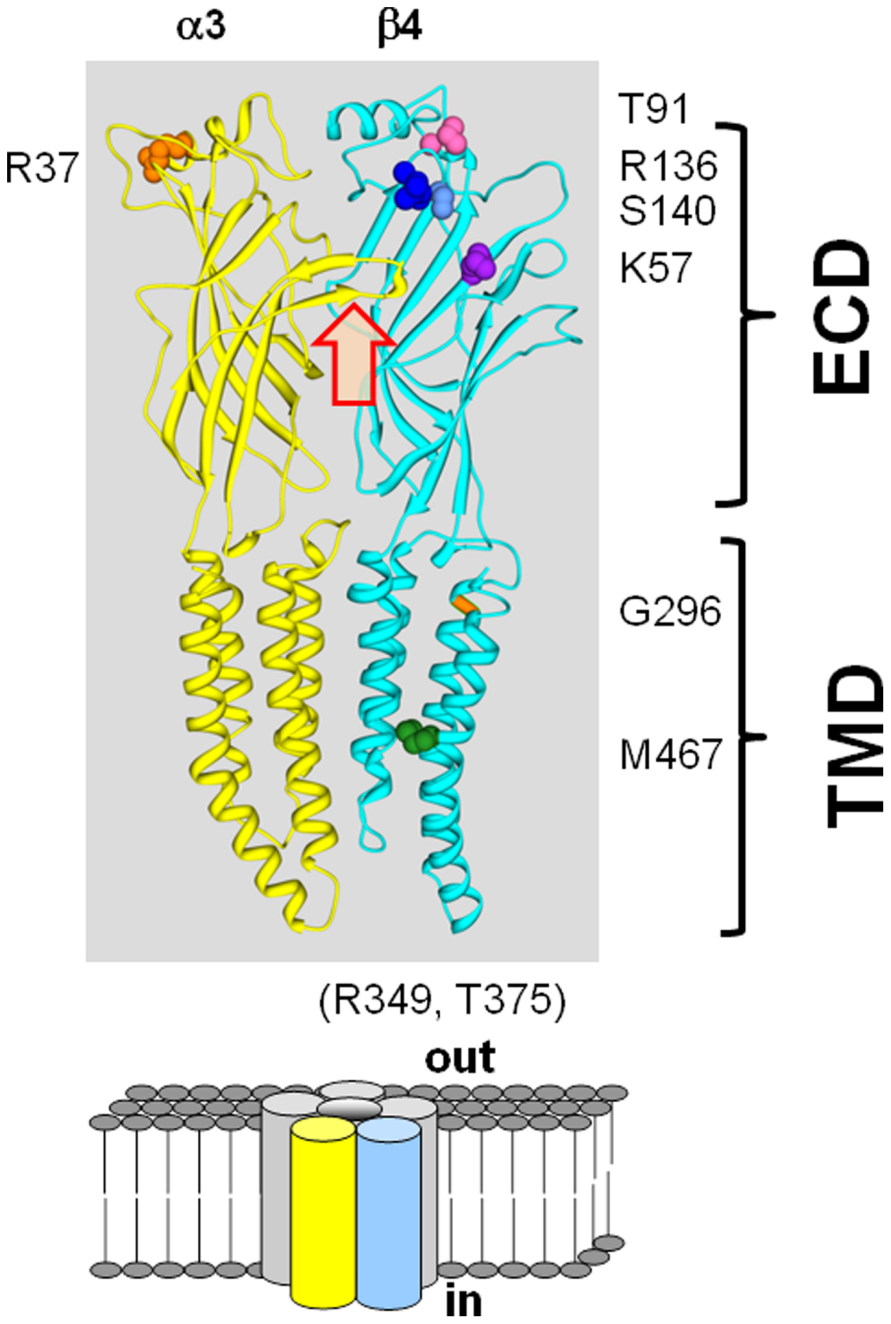
Locations of variants in a homology model of the α3β4 receptor. The figure shows a homology model of the α3 and β4 subunits, generated using the GluCl crystal structure (see Methods). The view is from the outside of the pentamer (see lower panel for cartoon), and to simplify the image the 3 subunits on the far side of the channel have been omitted. The backbones are shown in ribbon form (α3 yellow, β4 cyan). Locations of variants are shown as spheres colored as follows: in β4(T91) pink, K57 purple, R136 dark blue, S140 light blue, G296 orange, M467 dark green. A variant described in the α3 subunit (α3(R37)) is shown in orange on the α3 subunit. The location of the ACh-binding site is indicated by the red block arrow (the site is formed at the α3–β4 interface). The brackets at the right indicate the extracellular domain (ECD) and the transmembrane domain (TMD). Note that the large intracellular loop is not present in the crystal structure and so the β4(R349) and T375 residues are not shown.

The nicotinic receptor is a pentamer of highly homologous subunits [24]. Each subunit comprises a relatively large amino-terminal extracellular domain that contains the binding site for ACh, followed by a set of three closely spaced membrane-spanning helical domains (TM1–TM3) that contribute regions forming the ion channel. After these three domains is a cytoplasmic loop, containing sites for phosphorylation and for association with cytoplasmic proteins. The subunit ends with a 4th transmembrane helix and a short extracellular carboxy-terminal domain. Most variants are distributed in the N-terminal extracellular domain (Figure 1). None of the variants is predicted to be at contact regions between subunits, at which location they might affect inter-subunit interactions. Similarly, the variants are not located in the predicted ACh-binding regions, and none are located in the 2nd transmembrane domain that forms the major channel lining segment and likely contains the channel gate. The cytoplasmic domain is relatively poorly analyzed in terms of function and there is no information on its structure, so little interpretation can be made of variants in this region. In sum, there is no clear relationship between the location of the variants and their phenotype.

### Selection of β4 Variants

The goal of this study was to determine whether functional consequences of non-synonymous coding variants identified from deep resequencing of the *CHRNB4* locus could improve the association between genotype and behavioral phenotype (cigarettes smoked per day; CPD). Accordingly, we generated expression constructs containing the variants (see Table 1) and examined the abilities of these variant subunits to be expressed, to assemble into surface receptors and to function.

**Table 1:** *CHRNB4* missense variants examined.

Variant	PhyloP Score	Frequency (Number)	logCPD
β4	–	0.878 (1247)	0.01±0.03
β4(T91I)	2.16	0.006 (9)	−0.57±0.3
β4(R136Q)	0.41	0.002 (3)	0.13±0.07
β4(S140G)	2.27	0.022 (31)	0.09±0.17
β4(T375I)	2.26	0.014 (20)	−0.59±0.18
β4(M467V)	1.58	0.041 (58)	0.13±0.09
β4(M467V+R136W)	1.58	0.012 (17)	0.29±0.27
β4(M467V+S140G)	2.27	0.023 (32)	−0.11±0.13
β4(K57E)	1.16	0.001 (1)	0.31
β4(G296S)	5.69	0.001 (1)	−1.08
β4(R349C)	1.96	0.001 (1)	0.57

The first column shows variants in β4. The second column gives the PhyloP score for the variant. As discussed in Results, some alleles carried two variants (e.g. M467V+R136W). For alleles carrying two variants, the PhyloP score is the greater of the two separate variants. The column headed Frequency gives the frequency of the variant (number of chromosomes carrying the variant). The fourth column gives the measure of the behavioral phenotype used, the logarithm of the corrected cigarettes per day (see Methods; values given as mean ± SE). In the functionally weighted associations, singletons (bottom 3 rows) were weighted by the parameter value of the non-singleton variant or wild-type with the nearest parameter value to that of the singleton variant (Tables 2 and 3).

The β4 subunit can assemble with different α subunits to produce functional receptors. We focused on the properties of receptors containing the α3 and β4 subunits for several reasons. First, the genes encoding these subunits are located proximally in the human genome and have been shown previously to be highly correlated in expression patterns across brain regions [25]. Second, one rare non-synonymous variant in *CHRNA3* is in high linkage disequilibrium with a rare non-synonymous variant in *CHRNB4*, suggesting that there may be a functional relationship between these variants in human populations (see below). Lastly, this receptor subunit combination is expressed in the interpeduncular nucleus, the medial habenula and the ventral tegmental area, brain regions believed to be involved in addictive behavior, as well as being the dominant form expressed in peripheral ganglia [2]. We also expressed β4 subunits with the α4 subunit, as this α subunit is the most prevalent in the brain, and assembles with the β4 subunit in several brain regions [1].

A total of 10 rare missense variants were identified in *CHRNB4* and one rare missense variant was identified in the *CHRNA3* as part of the previous sequencing project. In this study we did not examine the properties of the variant *CHRNA3* subunit, so all experiments were done with wild-type α3 and α4 subunits. Analysis of the sequencing results reveals that several variants show linkage disequilibrium and thus cosegregate non-randomly in the population. Upon phasing of individual haplotypes, we observed that everyone (17 individuals) carrying β4(R136W) also carried β4(M467V) on the same allele, so we created an expression construct both β4(R136W) and β4(M467V) in the same subunit and used this double variant in functional studies rather than β4(R136W). In addition, a subset of individuals carrying β4(S140G) (32 out of 63) also carried β4(M467V) on the same allele. Accordingly, we created expression constructs containing both β4(S140G) and β4(M467V) and used this construct in studies, as well as β4(S140G) and β4(M467V).

### Subunit Expression: mRNA, Total Protein and Receptor Surface Expression

We first measured mRNA expression levels produced by plasmids containing each of the tested variants when expressed together with wild-type *CHRNA3* using qRT-PCR. We observed no significant differences between the mRNA levels produced from each variant-containing plasmid compared to wild-type *CHRNB4* containing plasmids (data not shown). These data suggest that any observed differences in total or cell-surface protein levels are not the result of altered initial mRNA levels.

Next, to determine if overall β4 protein, both intra-cellular and cell-surface, was altered by the introduction of variants, we performed western blots on total cell lysates from HEK 293 cells transiently transfected with wild-type α3 and either wild-type or mutant β4 containing plasmids. Analysis of the band intensity for each mutant protein provided no evidence of altered total β4 expression for any of the variant plasmids (data not shown). The S140G variant is predicted to abolish glycosylation at amino-acid position 138. The S is the third amino acid in the consensus glycosylation signal NXS, where X can be any amino acid. We hypothesized that the absence of glycosylation at this site should result in both faster migration on a western blot and low cell-surface protein expression. Both predictions were observed. Furthermore, treatment with PNGase to remove all glycosylation of wild-type β4 and β4(S140G) resulted in indistinguishable and more rapid migration (data not shown).

To determine whether mutations in *CHRNB4* alter the steady-state level of protein expressed on the cell-surface, we performed cell-surface ELISAs on cultured HEK 293 cells two days after transient transfection with constructs expressing either wild-type β4 or variant β4 in conjunction with wild-type α3 or wild-type α4. When the α3 or α4 subunits were transfected in the absence of a β4 subunit the surface ELISA signal was indistinguishable from that of cells transfected with empty vector (data not shown; two-tailed t-test P>0.9).

Cell-surface expression was decreased for nearly all variants tested (Tables 2 and 3). In addition, the correlation between surface expression with α3 and α4 subunits was high (adjusted r^2^ = 0.76, P = 0.01). The correlation suggests that the defect in surface expression results from some inability of the β4 variants to fold or traffic appropriately, rather than a specific defect in the ability to interact with a particular α subunit. Further experiments will be required to determine which mechanism(s) is more likely to explain the reduced surface expression for each variant (e.g. reduced assembly into pentamers, reduced forward trafficking to the plasma membrane or enhanced removal from the plasma membrane). An elegant study of a homologous mutation in the mouse β4 subunit (β4(R348C); [26]) reported that the presence of the variant reduced surface expression in cultured neurons by 2- to 3-fold (comparable to the 3- to 4-fold reduction in Tables 2 and 3) largely by reducing forward trafficking from the endoplasmic reticulum to the plasma membrane.

**Table 2:** Functional parameters for variants expressed with the α3 subunit.

Variant	Surface expression	ACh EC_50_ (µM)	Nic EC_50_ (µM)	Nicotine efficacy	Response to 1 µM Nicotine	“Sag” to ACh	“Sag” to nicotine
β4	1.00±0.00 (60)	146±34 (20)	22±4 (9)	0.73±0.03 (17)	0.011±0.003 (9)	0.86±0.02 (22)	0.86±0.02 (19)
β4(T91I)	0.27±0.06 (5)***	246±51 (7) NS	25±6 (8) NS	0.77±0.04 (15) NS	0.020±0.006 (8) NS	0.82±0.03 (13) NS	0.85±0.02 (11) NS
β4(R136Q)	0.16±0.16 (8)***	127±29 (6) NS	56±10 (5)*	0.69±0.05 (5) NS	0.011±0.003 (5) NS	0.88±0.02 (7) NS	0.82±0.05 (6) NS
β4(S140G)	−0.04±0.05 (12)***	121±20 (12) NS	48±4 (18)*	0.74±0.03 (18) NS	0.002±0.001 (13) NS	0.75±0.05 (12) NS	0.93±0.03 (15) NS
β4(T375I)	0.40±0.08 (5)***	227±77 (10) NS	33±12 (7) NS	0.81±0.04 (17) NS	0.032±0.010 (7) NS	0.84±0.03 (14) NS	0.91±0.02 (10) NS
β4(M467V)	0.73±0.22 (7)*	124±20 (12) NS	44±6 (11) NS	0.60±0.04 (11) NS	0.008±0.004 (10) NS	0.87±0.04 (13) NS	0.87±0.05 (15) NS
β4(M467V+R136W)	0.61±0.02 (2) NS	54±7 (16) NS	30±5 (17) NS	0.61±0.02 (17) NS	0.012±0.004 (17) NS	0.88±0.03 (14) NS	0.86±0.05 (17) NS
β4(M467V+S140G)	0.17±0.02 (2)***	163±37 (12) NS	18±3 (7) NS	0.63±0.06 (7) NS	0.013±0.003 (7) NS	0.90±0.03 (12) NS	1.00±0.01 (5) NS
β4(K57E)	−0.06±0.14 (6)***	110±23 (5) NS	62±11 (9)**	0.79±0.03 (18) NS	0.000±0.000 (9) NS	0.84±0.04 (4) NS	0.83±0.03 (9) NS
**(K57E)**	**(**−**0.04)**	**(121)**	**(56)**	**(0.77)**	**(0.002)**	**(0.86)**	**(0.82)**
β4(G296S)	0.97±0.29 (4) NS	57±38 (2) NS	19±3 (6) NS	0.74±0.05 (6) NS	0.020±0.009 (5) NS	0.94±0.03 (4) NS	0.98±0.01 (6) NS
**(G296S)**	**(1.00)**	**(54)**	**(18)**	**(0.74)**	**(0.02)**	**(0.90)**	**(1.00)**
β4(R349C)	0.24±0.05 (7)***	101±23 (5) NS	26±6 (4) NS	0.79±0.07 (10) NS	0.007±0.005 (4) NS	0.74±0.04 (10) NS	0.87±0.03 (9) NS
**(R349C)**	**(0.17)**	**(121)**	**(25)**	**(0.77)**	**(0.008)**	**(0.75)**	**(0.87)**

The first column shows the variant expressed. The remaining columns give the mean ± SE value for parameters, derived from (N) measurements. The values for singletons (variants occurring in a single individual) are shown below the empty row. In the functional weighting analysis these variants were grouped with the variant (or wild-type) parameter closest to the measured one (see Results); the values used are shown as bold font and in parentheses immediately below the measured parameter. Receptors containing α3 and β4(K57E) showed no response to 1 µM nicotine. For the variants, the notations give the significance of the difference to the wild-type value (one way ANOVA with Dunnett’s correction; NS P>0.05, *P<0.05, **P<0.01, ***P<0.001).

**Table 3:** Functional parameters for variants expressed with the α4 subunit.

Variant	Surface expression	ACh EC_50_ (µM)	Nic EC_50_ (µM)	Nicotine efficacy	Response to 0.3 µM Nicotine	“Sag” to ACh	“Sag” to nicotine
β4	1.00±0.00 (24)	9.36±1.35 (14)	1.48±0.17 (14)	1.00±0.03 (14)	0.059±0.012 (14)	0.90±0.02 (20)	0.91±0.03 (18)
β4(T91I)	0.31±0.05 (2)**	13.3±2.4 (8) NS	1.66±0.18 (8) NS	1.18±0.08 (8) NS	0.092±0.011 (8) NS	0.95±0.02 (12) NS	0.93±0.01 (12) NS
β4(R136Q)	0.88±0.25 (3) NS	5.97±1.42 (5) NS	2.01±0.31 (5) NS	1.06±0.08 (5) NS	0.122±0.019 (5) NS	0.26±0.06 (5)***	0.32±0.02 (4)***
β4(S140G)	−0.13±0.22 (2)***	16.7±2.1 (14)**	2.75±0.27 (13)**	0.96±0.05 (13) NS	0.022±0.003 (13) NS	0.76±0.04 (13)**	0.89±0.02 (13) NS
β4(T375I)	0.68±0.04 (2) NS	8.68±0.93 (7) NS	1.14±0.25 (7) NS	1.01±0.16 (7) NS	0.186±0.036 (7)***	0.76±0.04 (12)**	0.90±0.02 (11) NS
β4(M467V)	0.60±0.24 (4)*	8.24±0.75 (13) NS	1.40±0.11 (13) NS	1.07±0.06 (13) NS	0.044±0.006 (13) NS	0.85±0.02 (18) NS	0.92±0.02 (15) NS
β4(M467V+R136W)	nd	4.49±1.15 (7) NS	2.05±0.24 (6) NS	1.08±0.04 (6) NS	0.077±0.015 (6) NS	0.95±0.03 (7) NS	0.91±0.01 (6) NS
β4(M467V+S140G)	nd	12.5±1.7 (6) NS	1.56±0.24 (5) NS	0.88±0.07 (5) NS	0.035±0.013 (5) NS	0.90±0.03 (6) NS	0.79±0.10 (4) NS
β4(K57E)	0.03±0.03 (3)***	7.63±1.85 (4) NS	0.75±0.07 (9) NS	0.99±0.10 (9) NS	0.218±0.031 (9)***	0.93±0.01 (5) NS	0.84±0.03 (11) NS
**(K57E)**	**(**−**0.13)**	**(8.24)**	**(1.14)**	**(1.00)**	**(0.186)**	**(0.95)**	**(0.89)**
β4(G296S)	2.26±0.27 (6)***	nd	nd	nd	nd	nd	nd
**(G296S)**	**(1.00)**						
β4(R349C)	0.14±0.07 (4)***	6.31±0.92 (7) NS	1.36±0.04 (7) NS	1.01±0.04 (7) NS	0.063±0.008 (7) NS	0.88±0.02 (7) NS	0.99±0.02 (7) NS
**(R349C)**	**(0.31)**	**(5.97)**	**(1.40)**	**(1.01)**	**(0.059)**	**(0.90),**	**(0.93)**

The Table is presented in the same fashion as Table 2. For the variants, the notations give the significance of the difference to the wild-type value (one way ANOVA with Dunnett’s correction; NS P>0.05, *P<0.05, **P<0.01, ***P<0.001, nd: not done).

### Physiological and Pharmacological Studies of Variant Containing Receptors

To test the electrophysiological properties, receptors containing wild-type and variant β4 subunits were co-expressed with α subunits in HEK 293 cells. Whole-cell voltage-clamp experiments were performed to examine the basic functional properties of receptors containing variant subunits. Cells were selected using a bead-binding assay (see Methods) to increase our chance of obtaining measurable responses. To estimate the potency of ACh and nicotine we determined the concentration that produced a half-maximal response (EC_50_). A smaller EC_50_ value corresponds to a higher potency. To measure the relative efficacy of nicotine we determined the relative maximal response of a cell to nicotine compared to the maximal response to ACh. We also measured the relative response at the end of a 4 sec application of agonist, compared to the peak, to estimate the overall accumulation of receptors in a non-responsive (“desensitized”) state. Finally, we measured the relative response to a low concentration of nicotine (1 µM for α3-containing receptors and 0.3 µM for α4-containing receptors) to estimate the response to a more pharmacologically-relevant concentration of nicotine.

**Figure 2:**
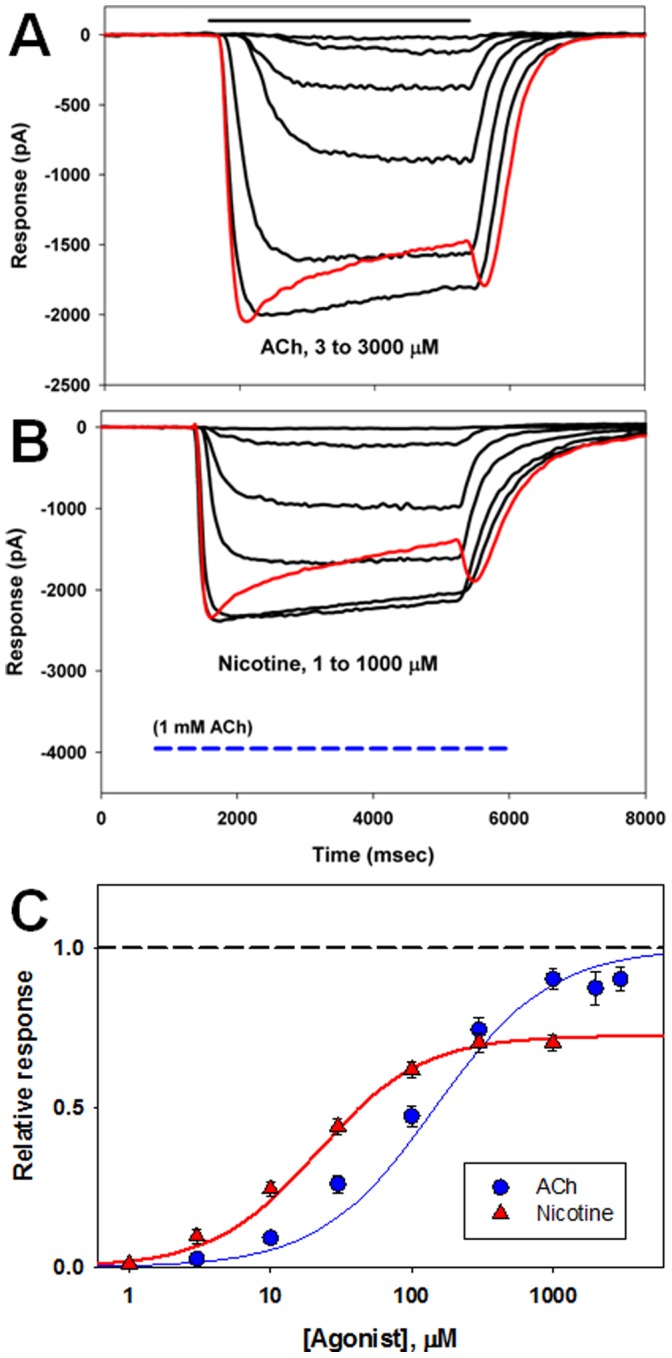
Activation of receptors containing α3 and wild-type β4 subunits. Panel A shows responses of a cell to various concentrations of ACh and Panel B shows the response of a different cell to various concentrations of nicotine (applications lasted 4 sec). In Panel B the horizontal dashed line indicates the response of this cell to 1 mM ACh, to estimate the maximal response. The response to the highest concentration of nicotine is clearly less than that to ACh, indicating that the relative efficacy of nicotine is less than that of ACh. Panel C shows the average normalized concentration effect curves for α3β4 receptors (both curves normalized to the maximal response for ACh). The symbols show mean ± SE. The curves show the predictions of the Hill equation (see Methods): Y(X) = a(1/(1+(X/EC50)n)) where a is the maximal response, X is the concentration of agonist, n is the Hill coefficient and EC50 is the concentration producing a half-maximal effect. The curves were generated with the overall mean parameters; for ACh EC50 146±34 µM and Hill coefficient 1.07±0.06 (20 cells), and for nicotine EC50 22±4 µM and Hill coefficient 1.17±0.07 (9 cells). The maximal response to nicotine relative to the maximal response to ACh (the relative efficacy for nicotine) is 0.73±0.03 (17 cells). The baseline holding current before application has been subtracted from all traces. The responses to the highest concentrations are shown in red, to emphasize the increased decline in response during the application and the rebound current when agonist is removed. This pattern is consistent with open-channel block by high concentrations of agonist.

Figure 2 shows typical physiological data and resulting concentration-response relationships. Agonist-induced open-channel block is present at the highest concentrations of agonist, as indicated by the rapid decline in response and the pronounced “rebound” response when agonist is suddenly removed (see red traces in Figure 2). Channel block was not studied further, as the physiologically relevant concentrations are either lower or much more transient.

**Figure 3:**
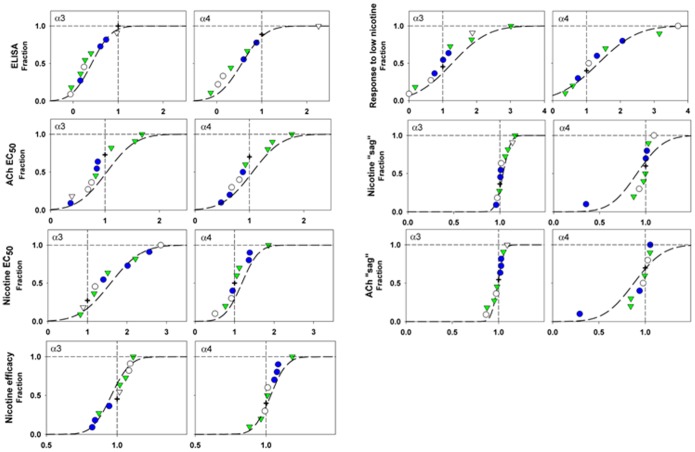
Consequences of variants for receptor properties. Each panel shows a cumulative distribution for the mean parameter values for receptors with different β4 variants. The left graph in each pair shows data for receptors containing the α3 subunit, and the right graph data for receptors containing the α4 subunit. The parameter values have been normalized to the value for wild-type β4 subunits to emphasize the relative functional changes (the actual data are shown in Tables 2 and 3). Data for receptors containing wild-type β4 are shown by plus signs, for β4 variants that have PhyloP scores less than 2 (“nonconserved”) by blue filled circles and for those with scores = >2 (“conserved”) by green filled inverted triangles. Hollow symbols show values for variants occurring in only single individuals; these values were not used in the association analyses (see Results). The vertical dashed line shows a relative value of 1 (equal to wild-type). The dashed curves show the cumulative normal distribution predicted by the mean and standard deviation of the data excluding singletons.

The results are summarized in Figure 3, displayed as the value for a variant-containing receptor relative to that for wild-type. This emphasizes the consequences on function for the variants. Measured data are presented in Tables 2 and 3.

We obtained concentration-response relationships for activation by ACh and nicotine. Graphs of typical relationships are shown in Figure 2 for α3β4 receptors. We estimated the potency of ACh and nicotine from the concentrations producing half-maximal activation (EC_50_), and results are summarized in Figure 3 and Tables 2 and 3. Few of the variants resulted in significant changes in the EC_50_ values for ACh (1 of 19 combinations) or nicotine (3 of 19).

The magnitude of the current elicited by a maximally effective concentration of agonist gives a measure of the efficacy of the agonist (that is, the maximal ability of the agonist to activate the receptor). We do not know this value in absolute terms, for example from single-channel studies that would directly measure the probability a channel is open. However, we can estimate the relative efficacy of nicotine compared to ACh from a ratio of the estimated maximal responses from a cell tested with both agonists. None of the combinations tested resulted in a significant change.

We obtained an estimate of the overall accumulation of receptors in non-responsive states from the amount of decrement (“sag”) from the peak response to the end of a 4 sec application of agonist. As discussed in Methods, we used responses elicited by an ∼EC_50_ concentration of agonist, to avoid possible complications due to channel block by high concentrations. A value of 1 for sag indicates that there was no decline in response, while a value of 0 indicates that the response declined completely to baseline. Most variants showed no significant difference from wild-type in sag. The β4(R136Q) variant showed greatly increased sag in the presence of either ACh or nicotine, but only when expressed with the α4 subunit.

Finally, we estimated the response to a low concentration of nicotine, as a fraction of the maximal response to ACh. The brain concentration of nicotine for a smoker is estimated to reach 100 to 1000 nM [27]. Receptors containing the α3 subunit did not reliably respond to nicotine concentrations less than 1 µM, while those containing the α4 subunit would respond reliably to 0.3 µM nicotine. Accordingly responses to these concentrations were used to estimate the relative response to a low concentration of nicotine. Few of the variants resulted in significant changes in this parameter (2 of 19 combinations).

In addition, we tested for correlations between parameters (e.g. ACh EC_50_ and nicotine EC_50_). Our motivation was to determine whether there were indications that variants had similar effects on functional responses to both ACh and nicotine (possibly suggesting an agonist-independent effect on activation) or on functional responses when expressed with the two α subunits. We did not correct the results for multiple comparisons (a total of 66). There was a significant association between response to low nicotine when expressed with an α3 subunit and response to low nicotine when expressed with an α4 subunit (r^2^ = 0.90; P = 0.001) and between the amount of sag when expressed with α3 and α4 (r^2^ = 0.87, P = 0.002). Overall, the functional effects of the variants did not fall into a simple pattern, such as both ACh and nicotine having reduced potency (increased EC_50_) when a variant is expressed with a given α subunit, nor a pattern in which similar effects occurred when a variant is expressed with different α subunits.

### Association Analyses

To determine if incorporation of results from our functional analyses of all variants known to exist in our study population could be used to improve power to detect an association between nicotine related behaviors and variants in *CHRNB4*, we created a genotype model weighted by the functional effects of each of the variants. We used parameters estimated for each variant for responses to nicotine and ACh as well as cell-surface protein level to create quantitative measures of receptor function that we could assign to each individual based on the alleles they harbor.

In order to compare our findings to conventional gene-based association methods, we first performed an analysis simply using carrier status at any non-synonymous site in *CHRNB4* as the predictor variable in a linear regression with log transformed lifetime maximum number of cigarettes smoked per day (logCPD) as the response variable and including age and sex as covariates. Using all missense variants, there is no significant association between carrier status at *CHRNB4* and logCPD (β = −0.04, p = 0.54, r^2^ = 0.0009) (Table 4). We have previously shown ([11]) that there is a significant association between carrier status of 4 variants (β4(T91I), β4(G296S), β4(T375I) and β4(M456V), each of these variants has a vertebrate phyloP score >2) and control phenotype (defined by FTND of < = 1). Accordingly, we selected only the subset of missense variants that occurred at genomic positions with an indication of cross-species conservation (defined by vertebrate phyloP scores >2; Table 1). This reduced the number of variants from 10 to 5. We observed a significant association between carrying at least one missense variant at a conserved site in *CHRNB4* and logCPD (β = −0.24, P = 0.01, r^2^ = 0.01) (Table 4). These observations provide a baseline for determining whether including experimental data on function improves the association of genotype with phenotype.

**Table 4:** Results from parameter-weighted linear regression.

Weighting	β	r^2^	P-value
Any Variant, unweighted	−0.04	0.0009	0.54
Variants with PhyloP>2	−0.22	0.008	0.01
Functional weighting			
α3 Low Nicotine	−0.29	0.021	6×10^−5^
α4 Low Nicotine	−0.25	0.016	4×10^−4^
α3 ACh EC_50_	−0.67	0.017	2×10^−4^
α4 ACh EC_50_	−0.12	0.0004	0.41
α3 Nicotine EC_50_	0.05	0.0003	0.26
α4 Nicotine EC_50_	0.22	0.001	0.16
α3 Nicotine Efficacy	−0.89	0.001	0.18
α4 Nicotine Efficacy	−0.11	0.001	0.86
α3 Cell-Surface Expression	0.053	0.0002	0.54
α4 Cell-Surface Expression	0.056	0.001	0.59
α3 ACh “sag”	0.27	0.0001	0.80
α4 ACh “sag”	0.25	0.0003	0.60
α3 Nicotine “sag”	−0.93	0.002	0.21
α4 Nicotine “sag”	0.08	.0001	0.89

The first column gives the weighting applied to the variant status. For each analysis, the phenotype used was log transformed CPD residuals (see Methods and mean values shown in Table 1). Carrier status was first used as an unweighted predictor of CPD, then carrier status at a subset of variant positions (those with phyloP scores >2; see Table 1). Lastly, carrier status was weighted by each of the listed parameters and used in the linear regression as the predictor. Variants of β4 were expressed with the α subunit shown, and parameter values used are in Tables 2 and 3). The columns give the slope of the relationship (β, where a negative value indicates that reduced CPD was associated with a larger value of the parameter), the adjusted r^2^ value and the probability that the slope is equal to zero. Associations were performed using linear regression in R.

**Figure 4:**
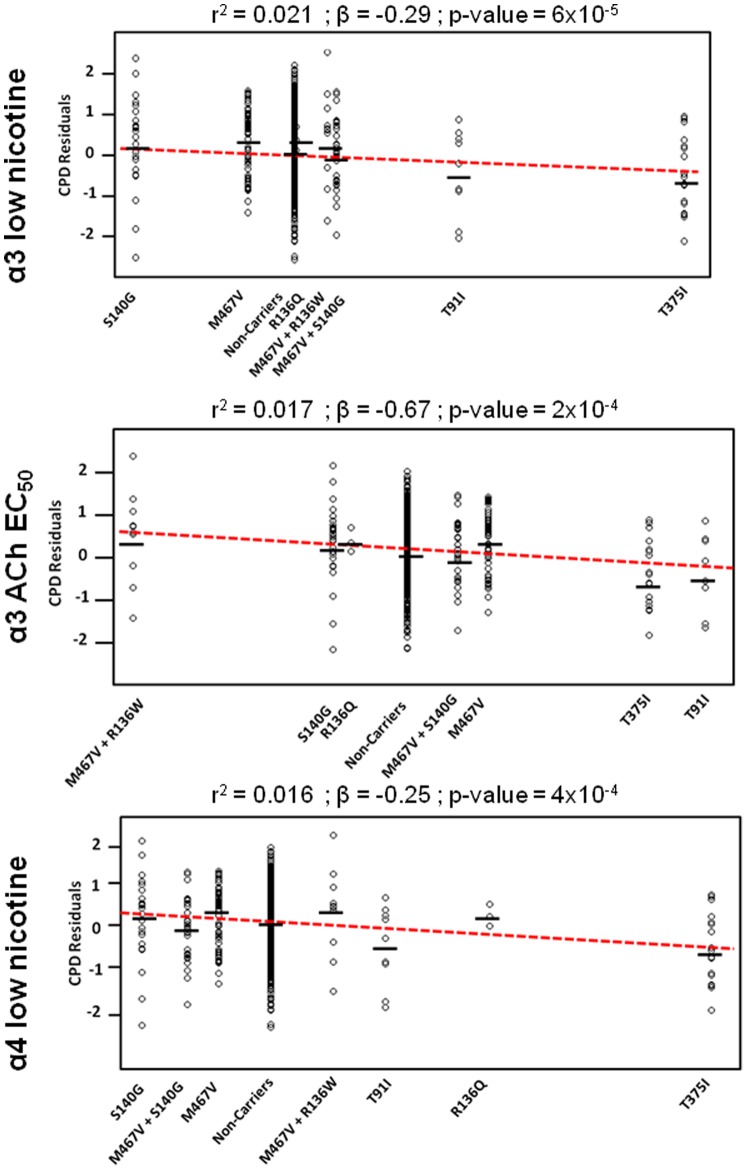
Correlation between Functional Parameters and CPD. The panels show the distribution of log(CPD) after correcting for age and sex, plotted against the relative parameter values for the 3 functional parameters that produced a significant association. The dashed red line is the estimated linear relationship. Horizontal lines mark the mean CPD residuals of carriers of each variant. Linear regression was performed in R. Singleton variants were included by weighting them by the parameter of the non-singleton variant (or wild-type) with the closest parameter estimate (see Results and Tables 2 and 3).

To perform parameter-weighted analyses of these data, we first had to decide which parameters to use for individuals harboring multiple variants. As mentioned, we observed that all individuals harboring β4(R136W) also harbored β4(M467V) on the same allele and that all individuals harboring both β4(S140G) and β4(M467V) harbored these variants on the same allele. As a result, we used parameters estimated from constructs harboring both variants. Every individual (a total of 9 in this population) carrying β4(T91I) also carried α3(R37H). However, we had elected to study properties of receptors with wild-type α subunits, and individuals with α3(R37H) also carried a wild-type α3 allele. We found (data not shown) that receptors containing the α3(R37H) variant expressed poorly on the cell surface and so it is likely that most functional receptors in the brain would contain wild-type α3 subunits. Finally, variants that occurred in only one individual were grouped with the non-singleton variant or wild-type with the nearest parameter estimate because values for the average CPD for singleton variants would be poorly estimated due to sample size (see Tables 2 and 3 for values used). We then weighted each variant genotype by the value for each of the estimated functional parameters. The results of the association analysis are summarized in Table 4. We find that weighting by ACh EC_50_ for variants expressed with α3 (β = −0.67, r^2^ = 0.017, P = 4×10^−4^) or by relative response to low nicotine when expressed with either α3 (β = −0.29, r^2^ = 0.021, P = 6×10^−5^) or α4 (β = −0.25, r^2^ = 0.016, P = 4×10^−4^) explained more phenotypic variance and produced a significant association. The results of this weighting procedure for these significant parameters can be seen in Figure 4.

It is interesting to consider why selecting variants based on PhyloP score improved the association, in the absence of any functional weighting. A possible explanation is suggested by considering Figure 3. It can be seen that the effects of variants at conserved locations (vertebrate PhyloP score >2) are concentrated at the right hand side of the distributions for the data for response to low nicotine (expressed with either α3 or α4) and for the ACh EC_50_ (expressed with α3). These parameters are the ones that resulted in significant improvement in the association when functional weighting was used, and the functionally-weighted association indicated that large values were protective. We then examined correlations between parameters and PhyloP score for all variants examined including singletons, using 2 metrics. The first was the relative parameter value, and the second was the absolute value of the logarithm of the relative parameter value. The second metric was used as a measure of effect size irrespective of direction, since relative values of 2-fold and one half would receive equal treatment. With a total of 32 regressions, 3 showed a P value less than 0.05: for “sag” for variants expressed with α3 the linear parameters had P = 0.015 and the logarithmic parameters P = 0.037, while for the ACh EC_50_ for variants expressed with α3 the logarithmic parameters had P = 0.039 (the linear parameters had P = 0.5) (results not corrected for multiple comparisons). These observations indicate that the PhyloP score is not a strong predictor of functional effect. Our observation that weighting variants based on occurrence at a conserved position improved association appears to be happenstance, and based on the particular distribution of functional effects with respect to PhyloP scores.

In order to determine if the association we observed between CPD and *CHRNB4* variants weighted by functional consequence could be due to chance, we performed permutation tests. For each of the significant parameters (response to low nicotine when expressed with α3, response to low nicotine when expressed with α4 or ACh EC_50_ when expressed with α3) we randomly assigned parameter values from the set of measured values we obtained to each of the variants without replacement and performed 10,000 or 20,000 permutations. The number of permutations performed was selected to provide a reasonable chance of obtaining a probability comparable to the experimentally observed one - for example with experimental P = 6×10^−5^, observing one more significant association in 20,000 permutations would give an estimate of 1/20,000 (5×10^−5^). In the case of response to low nicotine when expressed with α3 (association P of 6×10^−5^) none of 20,000 permutations had a lower P value, indicating that the association is likely significant at P<5×10^−5^. For ACh EC_50_ when expressed with α3 (P = 2×10^−4^) 48 of 10,000 had a lower P value, suggesting the association is likely significant at P<5×10^−3^. However, for response to low nicotine when expressed with α4 (P = 4×10^−4^) 960 of 10,000 permutations had a lower P value, suggesting that this association could readily arise by chance.

## Discussion

Common variants only account for a small fraction of the risk of developing nicotine dependence, suggesting that a major portion of the genetic contribution to the risk of developing nicotine dependence might result from many rare variants. We previously showed [11] that selected rare variants at 4 conserved residues in *CHRNB4* are associated with reduced nicotine dependence risk. We extended this analysis by including all variants and incorporating the experimentally determined functional consequences of the incorporation of subunits harboring rare variants. The mechanism underlying an association, however, can be unclear. The complexity of the effects of nicotine in the brain, its metabolism and the neuronal networks that lead to addiction make prediction of the relationship between genetic variants and behavior difficult. Even in the specific case of variants in the neuronal nicotinic receptors it is difficult to reach definitive conclusions regarding the role of variants in these genes *in vivo* in the absence of knowledge of the functional effects. This is largely due to two factors: (1) the role of neuronal nicotinic receptors in the brain is not well understood and (2) these receptor subunits combine in many different combinations, forming sub-types expressed in various patterns with indeterminate functional redundancy. Even when functional effects are identified, it can be difficult to directly connect the functional change to the behavioral phenotype and, at present, it is not possible to *a priori* associate a receptor functional phenotype with the risk of developing nicotine dependence. We have taken a different approach, which is to measure a number of basic functional properties and to determine which properties improve the association between genotype and phenotype, with the idea that this will provide insights into possible underlying functional changes.

Accordingly, we characterized the functional consequences of rare variants in the *CHRNB4* gene observed from sequencing a cohort of nicotine dependent individuals and non-dependent smoking controls. These parameters were then used to weight variants in a gene-based association test to test the hypothesis that relevant parameters will improve the observed association at this locus.

The majority of variants significantly reduced cell-surface protein expression. Despite this, cell-surface expression was not significantly associated with CPD in this dataset. This is, perhaps, expected as mice lacking the β4 subunit only show alterations in nicotine withdrawal symptoms and decreased adverse effects at high nicotine doses compared to wild-type, suggesting that even large effects on protein level may have small effects on behavior [28]. The overall consequences in the brain of reduced surface expression will depend on several factors, as the variant occurs in the presence of a wild-type allele. The effects will likely depend on the exact mechanism underlying the reduction, which our data do not address, and the overall metabolism of the receptors and subunits. If subunit maturation of the variant subunit is reduced so that it does not even assemble to form a pentameric receptor, then it appears likely that the majority of surface receptors will contain the wild-type allele, albeit at a possibly reduced level from a homozygous wild-type individual. The amount of reduction will depend on whether the β4 subunit is normally in excess in comparison to other subunits. If, on the other hand, the variant subunit assembles but reduces forward trafficking (or increases retrieval from the plasma membrane) of pentamers then it seems likely that some fraction of surface receptors will contain a variant subunit because the variant will be incorporated into pentamers and susceptible to trafficking to the surface. The proportion will depend on the stoichiometry of the assembled pentamers and on the effects on trafficking resulting from incorporation of 1, 2 or 3 variant subunits. However, likely only a minority of receptors would contain only wild-type subunits. Given this uncertainty, the lack of major effects of gene knockout in mice, and the lack of improved association when surface expression was used as a single functional weighting parameter, we did not pursue multiple factor association analysis incorporating data on surface expression.

Most variants had relatively small effects on the aspects of receptor function we measured. However, even in the face of these small individual effects we found that 3 parameters provided large increases in the significance of the association between genotype and phenotype. Our results indicate that among the parameters estimated for each of the *CHRNB4* variants, response to low concentrations of nicotine and ACh EC_50_ were able to improve the observed association between rare variants in *CHRNB4* and CPD. In each case the association indicated that large values of the parameter is protective - a larger response to low nicotine or a less potent effect of ACh. This suggests that overall an enhanced response to concentrations of nicotine that may be reached in the brain of a smoker may be a protective factor. The other parameters did not result in significant improvements in association, although the overall trends (Table 4) were that decreased nicotine EC_50_, increased nicotine efficacy or increased ACh EC_50_ were associated with reduced CPD. Each of these trends would be associated with a larger relative response to low nicotine, everything else being equal.

There are some experimental factors in our approach that affect the interpretation of these results. First, all experiments were performed in HEK 293 cells. This was done for several reasons. The major one is that it allowed us to define the subunit composition of the expressed receptors, and hence to examine consequences of expression of variants with different α subunits. A second reason is that it provided a robust expression system that would be less susceptible to variability due to phenotypic diversity, including endogenous subunit expression, in neuronal cells. Finally, HEK 293 cells are widely used and therefore are suitable to replication or extension of our work by other laboratories. However, these experimental advantages come with a significant simplification of the situation in the brain. In particular all neuron-specific effects on assembly, trafficking or post-translational modification are lost in this model. The use of single α and β4 subunits does not recapitulate the natural competition among subunits for incorporation into receptors in neurons, which typically express multiple subunits. Our approach also presumes that the effects we see when *CHRNB4* variants are expressed with the α3 or α4 subunits are the most informative of the possible subunit combinations. These α subunits were chosen based on prevalence and possible relevance to nicotine behavioral phenotype, but it may be that other combinations are more directly involved in nicotine dependence. Indeed, the lack of correlations between physiological consequences when variants were expressed with the α3 compared to α4 subunits suggests that the variants may have different effects in receptors of different composition. Finally, only a limited number of functional parameters were assayed, and it may be that the most significant attribute was missed.

A further caveat to our interpretations arises from the observation that αβ nicotinic receptors can assemble in 2 subunit stoichiometries - 2α to 3β or 3α to 2β - with different properties. Studies of α3β4 and α4β4 receptors indicate that both of these subunit combinations can assemble in either stoichiometry [29], [30]. However, we used only a single subunit stoichiometry in our transfections. This caveat is given particular significance by the recent report that rare variants in the α4 subunit can result in changes in subunit stoichiometry for α4β2 receptors [31]. It is possible that similar effects might occur with the β4 variants we tested, and thereby produce some of the alterations in functional properties.

Few studies have been made of the functional properties of receptors containing variant β4 subunits. In one, 4 variants were expressed in *Xenopus* oocytes with the α4 subunit [32]. The authors tested the T91I, R136W, S140G and M467V variants, and found relatively small changes in the EC_50_ for ACh of a similar magnitude and direction to our observations. The second study examined the properties of the R349C variant, expressed in GH4C1 cells [33]. In this study, a relatively large increase in the EC_50_ for ACh was found when the variant was expressed with the α3 subunit (3.2-fold) in comparison to our modest decrease (to 0.7-fold). When expressed with the α4 subunit, the variant increased the EC_50_ for ACh by 3.3-fold (compared to a decrease to 0.7 in our results) and that for nicotine by almost 17-fold (compared to a decrease to 0.9). However, there did not appear to be any change in nicotine relative efficacy (as we also found). We do not have an explanation for the difference in results.

Recent work suggests that the α3β4* subtype of nicotinic receptors may contribute significantly to nicotine related behaviors by activating the habenulo-interpeduncular pathway [25], [34], [35]. Additionally, a recent association study of smoking cessation has shown that variants in *CHRNB4* may decrease craving and withdrawal symptoms [36]. Our results are consistent with these reports in indicating a role for the β4 subunit in smoking behavior.

The single variant with the strongest association with nicotine dependence is a nonsynonymous coding variant in the α5 subunit, which results in the replacement of aspartate 398 with asparagine [6]. This variant has been found to enhance desensitization [37] and reduce the maximal response to agonists [38], and is associated with an increased risk of nicotine dependence. A very recent study found that three rare variants in the major cytoplasmic loop of the α4 subunit affected several properties of α4-β2 receptors, including enhancing the proportion of receptors that showed a high sensitivity to ACh [31]. These variants are found more often in control than nicotine dependent individuals [10]. Finally, studies of a variant in the α4 subunit in mice have found that it increases the sensitivity to nicotinic activation and also reduces the severity of responses to nicotine [39]. These observations have led to the suggestion that reduced sensitivity to activation by agonists results in increased risk for developing nicotine dependence and that, conversely, increased sensitivity reduces the risk [31]. Our results agree with this conclusion, and extend it by implicating an increased relative sensitivity to activation by nicotine as the critical factor.

The present work suggests that future analysis of rare variant associations may depend on the development of high-throughput methods of assessing allele function in order to clearly distinguish the alleles with functional effects. This is due largely to three factors. First, genes are capable of harboring alleles with opposing effects and these opposing effects obscure each other in a collapsing approach. For instance, in our data, the T375I variant is protective (lower average logCPD) while the S140G variant is a risk allele, although both have PhyloP score >2. Second, alleles that alter protein function do not necessarily have the same magnitude of effect and as such the variants of lesser effect can obscure the variants of greater effect, particularly if they are of higher frequency. This was the case for the M467V+S140G and T375I variants. Both are protective alleles, but the M467V+S140G variant confers only slightly lower risk than normal while the T375I confers much less risk. Lastly, one technique to gain power is to use only variants with putative higher prior probability of having effects on the function of the gene product (i.e. missense or nonsense variants, variants at evolutionarily conserved sites). This often greatly reduces the number of variants and the number of carriers available for association testing, thereby reducing power and possibly obscuring associations if variants have opposing effects. When all variants are assessed for function, each can be used in the association test regardless of magnitude or direction of effect.

Overall, our results indicate that incorporating functional information into association analyses can improve power to detect associations if relevant parameters are measured, and that methods of assaying the functional impact of variants across the genome will likely greatly improve our ability to understand the genetic basis for diseases in the era of whole-exome and whole-genome sequencing. The reduction in power resulting from large proportions of variants with little or no impact on protein function or mixtures of protective and risk variants being included in gene-based burden tests is substantial and will have to be addressed if we hope to understand the full scope of variation impacting complex diseases and traits. The results can also suggest possible mechanisms for an association, in the present case indicating that a larger relative response to low concentrations of nicotine may reduce the risk of developing nicotine dependence.
